# Performance analysis of CRF-based learning for processing WoT application requests expressed in natural language

**DOI:** 10.1186/s40064-016-3012-9

**Published:** 2016-08-11

**Authors:** Young Yoon

**Affiliations:** Department of Computer Engineering, Hongik University, 94, Wowsan-ro, Mapo-gu, Seoul, South Korea

**Keywords:** Web of Things, Natural language processing, Conditional random fields, Application composition

## Abstract

**Background:**

In this paper, we investigate the effectiveness of a CRF-based learning method for identifying necessary Web of Things (WoT) application components that would satisfy the users’ requests issued in natural language. For instance, a user request such as “archive all sports breaking news” can be satisfied by composing a WoT application that consists of ESPN breaking news service and Dropbox as a storage service.

**Findings:**

We built an engine that can identify the necessary application components by recognizing a main act (MA) or named entities (NEs) from a given request. We trained this engine with the descriptions of WoT applications (called recipes) that were collected from IFTTT WoT platform. IFTTT hosts over 300 WoT entities that offer thousands of functions referred to as triggers and actions. There are more than 270,000 publicly-available recipes composed with those functions by real users. Therefore, the set of these recipes is well-qualified for the training of our MA and NE recognition engine.

**Conlusions:**

We share our unique experience of generating the training and test set from these recipe descriptions and assess the performance of the CRF-based language method. Based on the performance evaluation, we introduce further research directions.

## Background

IFTTT is a platform that hosts Web of Things (WoT) entities that are referred to as channels.^1^ These channels offer functionalities such as triggers and actions which are ingredients for event-driven applications called recipes. For example, a user can manually compose an application that consists of ESPN breaking news service as a trigger and a text-messaging service as an action. Once the user activates this application, the user will start to receive text notifications whenever any ESPN breaking news gets published. In Hyun et al. (2015), we presented the ultimate goal of enhancing user experience by demonstrating a conceptual system that automatically composes and executes an IFTTT recipe given a user request issued entirely in natural language.

However, this system fell short in correctly identifying the intention behind the requests that are oftentimes ambiguous and irregular. For instance, suppose a user issues a request such as *“Let me know whenever any breaking news in sports gets published”*. In this request, the exact news source is not specified, and the request can be expressed quite differently such as *“If I receive a breaking sports news, notify me”*. It was also difficult for our system to recognize which parts of the sentence relates to a desired trigger or an action. This shortcoming prompted us to investigate the feasibility of devising an engine that can learn what triggers and actions are actually asked for in the requests issued in natural language. Specifically, we employ a CRF-based learning method (Dafferty et al. 2001) that has been successful in natural language processing (NLP) operations such as part-of-speech (POS) tagging and named entity recognition (NER). The details of the learning procedure is presented in the following section.

## Methods

In this section, we first present a CRF-based learning framework. Then, we explain training set generation and evaluation methods.

### The learning framework

**Fig. 1:**
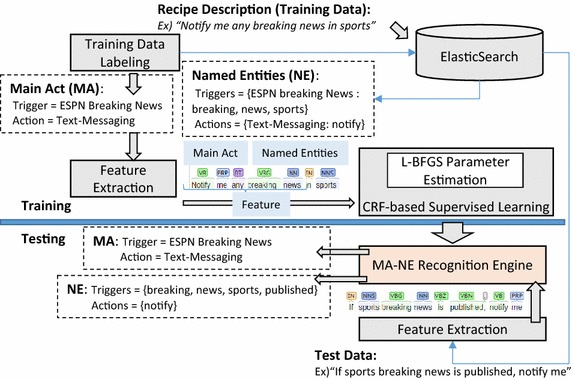
Illustration of the CRF-based IFTTT recipe description learning framework

In Fig. 1, we illustrate the overall framework for learning the best-matching IFTTT triggers and actions for user requests. We explain the supervised learning procedure as follows. We collect recipe descriptions and label them with either a main act or named entities so that they can be used as training data. A main act (MA) is a sequence of a trigger ID and an action ID, which is associated with the whole recipe description. A named entity (NE) is either a trigger ID or an action ID. Each noun or verb in a recipe description is labeled with a named entity. After the labeling, we extract the feature of the recipe description which is a sequence of POS tagged tokens. We used Stanford NLP for POS tagging (Kristina et al. 2003). The feature set along with the MA and NE labels is fed into the CRF-based learning engine (Dafferty et al. 2001). We used L-BFGS parameter estimation algorithm (Andrew and Gao 2007) in order to iteratively search for a function that can find the most-likely correlation between features and MAs/NEs. This function is used in the engine that recognizes a MA and NEs of a new given recipe description. As the first step of testing the MA and NE recognition engine, we POS tag every recipe description in a test set to extract features. Given a feature as an input, the MA and NE recognition engine outputs a MA or a sequence of NE-labeled tokens.

### Training & testing and evaluation method

We collected more than 270,000 publicly available recipe web pages from IFTTT in a non-invasive way using Crawler4J.^2^ Every recipe web page has a description of the recipe and the IDs of the trigger and the action that were actually used for the recipe. We scrapped these information all together using JSoup^3^ and Selenium^4^ and then stored them into ElasticSearch^5^ as a single document. We randomly sampled 1000–9000 recipe descriptions according to uniform distribution, and labeled them with MAs and NEs so that these can be used as training data. Labeling each verb and noun in the recipe description with a NE was challenging, because the recipe information does not tell exactly which verb or noun in the description is associated with which trigger ID or action ID. Instead of manually labeling the tokens with a NE, we exploited the search functionality of ElasticSearch as follows. We match a token in a recipe description against the two sets of documents in ElasticSearch, one indexed by the trigger ID and the other indexed by the action ID. We picked a set that retrieves documents with higher average relevancy score. Then we labeled the given token with the index (trigger ID or action ID) of the selected document set.

We generated two sets of randomly selected recipes, in order to test the effectiveness of our MA and NE recognition engine that was trained with the aforementioned training set. One test set contains 200 recipes, and the other contains 7000 recipes. Note that we only included recipe descriptions expressed in English. We excluded recipe descriptions that contain jargons that were not recognizable by Stanford NLP. We also excluded any recipe description that contained less than 2 words, as it would be too terse to convey any information. Some of the recipes under popular channels such as Facebook contained meaningless advertisements in the recipe description, and these were ruled out as well. This rigorous filtering was necessary to control the quality of the training and test data.

Training and testing were conducted on an Ubuntu 14.04 server with Intel i5 3.2 GHz CPU and 4 GB of memory. We measured how accurately our recognition engine can identify the trigger ID and the action ID. If the engine correctly yields both trigger ID and action ID then its accuracy is 100 % for the given test recipe description. If only one ID is correctly identified, the accuracy is 50 %, and if no correct ID is identified then its accuracy is 0 %. We computed the average accuracy of all test data. We also measured the time it took to train the recognition engine. We provide the analysis of the evaluation results in the following section.

## Results and discussions

As shown in Table 1, the MA recognition was more effective than the NE recognition in identifying the correct trigger ID and action ID. NE recognition showed accuracy up to 32.7 % which is 57 % less than the maximum accuracy of MA recognition. The poor performance of NE recognition is due to imperfect NE labeling which was done automatically using ElasticSearch. Also, we could not account for any context of a token that we labeled with a NE. For instance, the word “light” can be used in a condition phrase such as *“If light is turned on”*, or in an action phrase such as *“turn on the light”*. Since our method is context-aware, the token “light” can be labeled with a wrong NE tag. We plan to employ techniques for phrase-level tokenization and grammar parsing in order to improve accuracy of identifying a correct trigger or action for a given phrase.

**Table 1:** Accuracy (%) MA and NE recognition

Size of training data	MA recognition		NE recognition	
	Test set		Test set	
	200	7000	200	7000
1000	38.8	41.4	28.1	26.9
3000	42.8	45.8	29.1	29.9
5000	49.3	51.1	29.4	31.3
7000	42.0	51.3	29.4	30.2
9000	44.5	51.6	32.8	32.7

MA recognition is relatively more promising in identifying triggers and actions

Although the MA recognition seems relatively more promising, we observed little gain in the accuracy when the training set size increased to more than 5000 recipe descriptions. This was due to the characteristics of the training set that a small set of triggers and actions were used frequently in the recipes. In fact, top-10 triggers and actions were used in up to 75 and 48 % of the recipes in the training set. This biased learning actually caused an overfitting problem. To remove the bias, we collected the same number of recipe descriptions per trigger and action. However, the training data collection method worsen the accuracy of MA recognition. It turned out that the number of features to learn a MA was too small.

As shown in Table 2, the training time was excessively long especially when there were a large number of features to learn. Despite the lengthy training time, only a small fraction of MAs or NEs were learned. There are ${\text{m}} \times {\text{n}}$ MAs, where ${\text{m}}$ and ${\text{n}}$ are the numbers of triggers and actions, respectively. Learning all possible MAs is infeasible considering the fact that there are currently over 2000 triggers and actions. Learning NEs would be more feasible as the total number of NEs equal to the number of triggers plus the number of actions. However, as mentioned above, we have to first resolve the issue with the imperfect NE labeling during the automatic generation of training data. Also, the current approach of attempting to learn all MAs and NEs all at once is impractical, as the number of things (channels) hosted on IFTTT is constantly growing. Therefore, at any time the MA and NE recognition engine created through our framework can become obsolete.

**Table 2:** Training time (min.) and the number of learned MAs, NEs and features MAs and NEs are insufficiently learned despite the long training time

Size of training data	MA recognition			NE recognition		
	Test set			Test set		
	# of MAs	# of features	Training time	# of MAs	# of features	Training time
1000	392	1453	0.4	117	28,715	1.3
3000	773	3302	51.3	138	699,932	186.5
5000	1139	4702	340	180	108,099	745.3
7000	1494	6326	586.6	200	152,983	1123.2
9000	1839	7704	962.2	231	203,145	2100.7

MAs and NEs are insufficiently learned despite the long training time

We plan to revise the current learning framework as follows. Instead of randomly sampling recipe descriptions, we can group recipe descriptions by channels. We train each group and create a separate MA and NE recognition engine per channel. Given a new request, we first select the most relevant channel and then query the associated MA and NE recognition engine to identify triggers and actions that would satisfy the request. We expect this two-phase approach to improve the accuracy. In addition, we can reduce the training time by parallelizing the procedure of creating the MA and NE recognition engine per channel. Furthermore, we can incrementally generate a separate MA and NE recognition engine for a newly introduced channel without changing other recognition engines.

## Conclusions

We devised a CRF-based learning framework to generate an engine that can recognize desired triggers and actions for user requests specified in natural language. We created training data from a set of carefully selected publicly-available IFTTT recipe descriptions. Given the training data, the CRF-based learning engine takes the POS-tagged tokens in the recipe descriptions as features and learned a main act (a pair of trigger and action) for a whole recipe description and named entities (a trigger or an action) for every token in a recipe description. The MA recognition approach was more promising in finding the desired triggers and actions compared to the NE recognition approach. However, both MAs and NEs were insufficiently learned despite excessive training time. Considering the excessive training time and the fact that the number of things (channels) hosted on IFTTT is constantly growing, we cannot recommend the currently approach of learning all MAs and NEs all at once. As a future work, we plan to devise a framework that allows parallel and incremental learning that can achieve higher accuracy and reduce learning time.

## Footnotes

1 IFTTT Web of Things Application Platform. http://www.ifttt.com.

2 Crawler4J. https://github.com/yasserg/crawler4j.

3 Jsoup, Java HTML Parser. http://jsoup.org/.

4 Selenium, Web Brower Automation. https://github.com/yasserg/crawler4j.

5 ElasticSearch. https://www.elastic.co/products/elasticsearch.
